# Enterobius vermicularis as an Intraoperative Surprise in a Child With Suspected Appendicitis and Normal Laboratory Findings

**DOI:** 10.7759/cureus.94036

**Published:** 2025-10-07

**Authors:** Younis Al-Mufargi, Khadija Al Musalhi, Moayed Khalil, Mostafa Hamad, Abdulmalik Al-Atar

**Affiliations:** 1 General Surgery, Medical City for Military and Security Services, Muscat, OMN; 2 General Surgery, Armed Forces Hospital, Muscat, OMN; 3 Pediatric Surgery, Medical City for Military and Security Services, Muscat, OMN

**Keywords:** appendicitis, case report, enterobius vermicularis, pediatric surgery, pinworm

## Abstract

Acute appendicitis is a common surgical emergency in children, typically associated with elevated inflammatory markers and classical clinical presentation. However, parasitic infections such as *Enterobius vermicularis* can occasionally mimic appendicitis and represent a diagnostic challenge. Herein, we report a case of a nine-year-old female child who presented with right iliac fossa (RIP) abdominal pain and vomiting with normal white blood cell count and C-reactive protein (CRP) levels. Ultrasound imaging suggested early appendicitis, and therefore, a laparoscopic appendectomy was performed. Intraoperatively, multiple *E. vermicularis* worms were found within the appendiceal stump. Histopathological examination revealed no acute inflammation, but confirmed the presence of the *E. vermicularis* in the lumen. The patient recovered uneventfully and received antihelminthic therapy with albendazole. This case highlights the importance of considering parasitic infections in the differential diagnosis of appendicitis, particularly in pediatric patients with atypical presentations. It also emphasizes the diagnostic value of intraoperative findings and histopathology in guiding postoperative management.

## Introduction

Acute appendicitis is a common surgical emergency, often caused by luminal obstruction due to fecaliths, lymphoid hyperplasia, or foreign bodies [1]. However, parasitic infections, particularly those caused by *Enterobius vermicularis*, commonly known as pinworms, represent an uncommon yet noteworthy etiology of appendiceal inflammation [2].

Among parasitic infections, *E. vermicularis* is the most widespread helminthic infection worldwide, especially prevalent among children aged five to 14 years [3]. The parasite is transmitted via the fecal-oral route, with female worms depositing eggs around the perianal region, typically resulting in nocturnal anal pruritus. While pinworm infection usually presents with benign and localized symptoms, its presence in the appendix has been associated with appendiceal pathology, although the precise pathogenic role remains controversial [4,5].

Diagnosing appendicitis secondary to* E. vermicularis* poses a clinical challenge. The clinical picture often mimics that of classical acute appendicitis, but inflammatory markers such as leukocyte count and C-reactive protein (CRP) may remain within normal limits [6]. Imaging modalities are often inconclusive, and in most cases, the definitive diagnosis is made intraoperatively or during histopathological examination of the resected appendix [7]. In some cases, the parasite is considered an incidental finding; in others, it is implicated as a causative agent in the inflammatory process [8]. While previous studies have debated the pathogenic role of* E. vermicularis*, few reports focus on cases where laboratory and imaging findings are inconclusive, underscoring the diagnostic uncertainty in such presentations. This report highlights an uncommon presentation of *E. vermicularis* in the appendix of a child with normal inflammatory markers, contributing to the ongoing debate about its role in appendiceal pathology.

## Case presentation

A nine-year-old female with no significant past medical or surgical history presented to the Emergency Department (ED) in our hospital with a three-day history of progressively worsening abdominal pain. The pain was described as dull, initially periumbilical, and radiating to the lower abdomen, with increased severity at night and exacerbated by movement. The abdominal pain was associated with nausea, anorexia, and multiple episodes of non-bilious vomiting (three to four times on the day prior to admission). She also reported a single episode of diarrhea on the morning of presentation. There were no urinary symptoms.

In the ED, the patient received intravenous analgesia and antiemetics, which resulted in symptomatic relief. On physical examination, the abdomen was soft, with localized tenderness in the right iliac fossa (RIF) and suprapubic region. Rebound tenderness was present in the RIF. However, Rovsing’s sign was negative. Bowel sounds were audible, and both respiratory and cardiovascular examinations were unremarkable.

The patient was afebrile and hemodynamically within normal on arrival. Vital signs were as follows: temperature, 36.8°C; heart rate, 100 bpm; and blood pressure, 100/70 mmHg. Laboratory tests were within normal limits, including white blood cell count and serum CRP level, as shown in Table 1. Liver function tests, urea, and electrolytes were within normal limits.

**Table 1:** Laboratory evaluation during admission CRP: C-reactive protein

Laboratory test	Patient’s value	Normal range
CRP serum	1.5	0.0-5.0 mg/L
WBC count	4.44	(4.50-14.50)×10⁹/L
Red blood cells	5.89	(3.90-5.30)×10^12^/L
Hemoglobin	12.2	11.5-15.5 g/dL
Platelet count	372	(150-450)×10⁹/L
Neutrophils	1.6	(1.4-9.0)×10⁹/L
Lymphocytes	1.9	(1.9-9.8)×10⁹/L
Monocytes	0.3	(0.1-1.0)×10⁹/L
Eosinophils	0.4	(0.1-0.8)×10⁹/L
Basophils	0.0	(0.0-0.2)×10⁹/L

Despite the clinical suspicion of appendicitis, laboratory findings, including normal CRP and borderline low WBC, did not support significant systemic inflammation, complicating the preoperative diagnosis.

**Figure 1:**
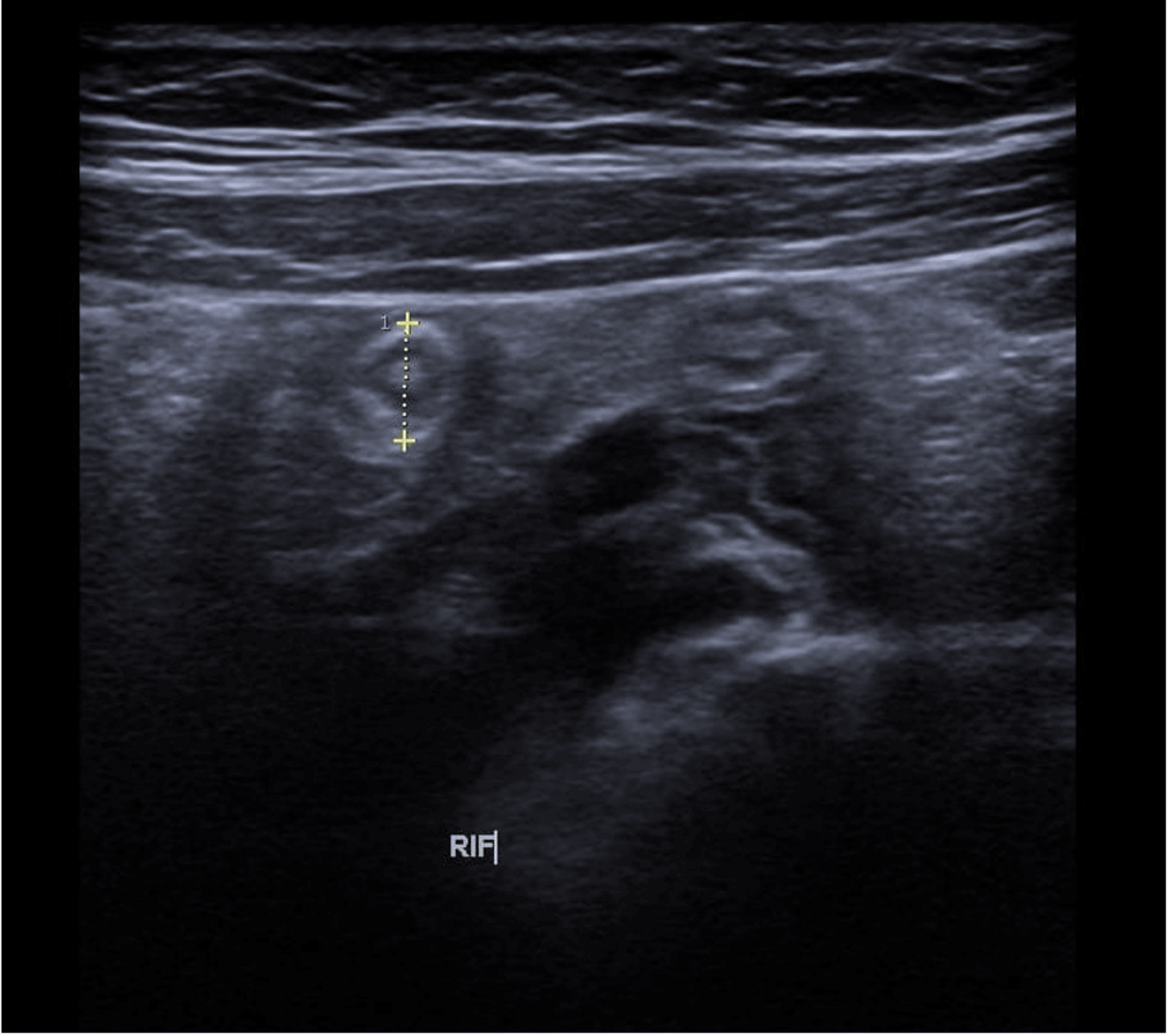
Abdominal ultrasonography demonstrating a non-compressible, dilated appendix (6 mm diameter) located in the RIF, consistent with early acute appendicitis RIF: right iliac fossa

Abdominal ultrasonography revealed a non-compressible and dilated appendix located in the RIF, anterior to the right iliac vessels (Figure 1). The appendix measured 6 mm at the base and 5 mm at the tip, with mild probe tenderness and adjacent free fluid findings consistent with early acute appendicitis.

**Figure 2:**
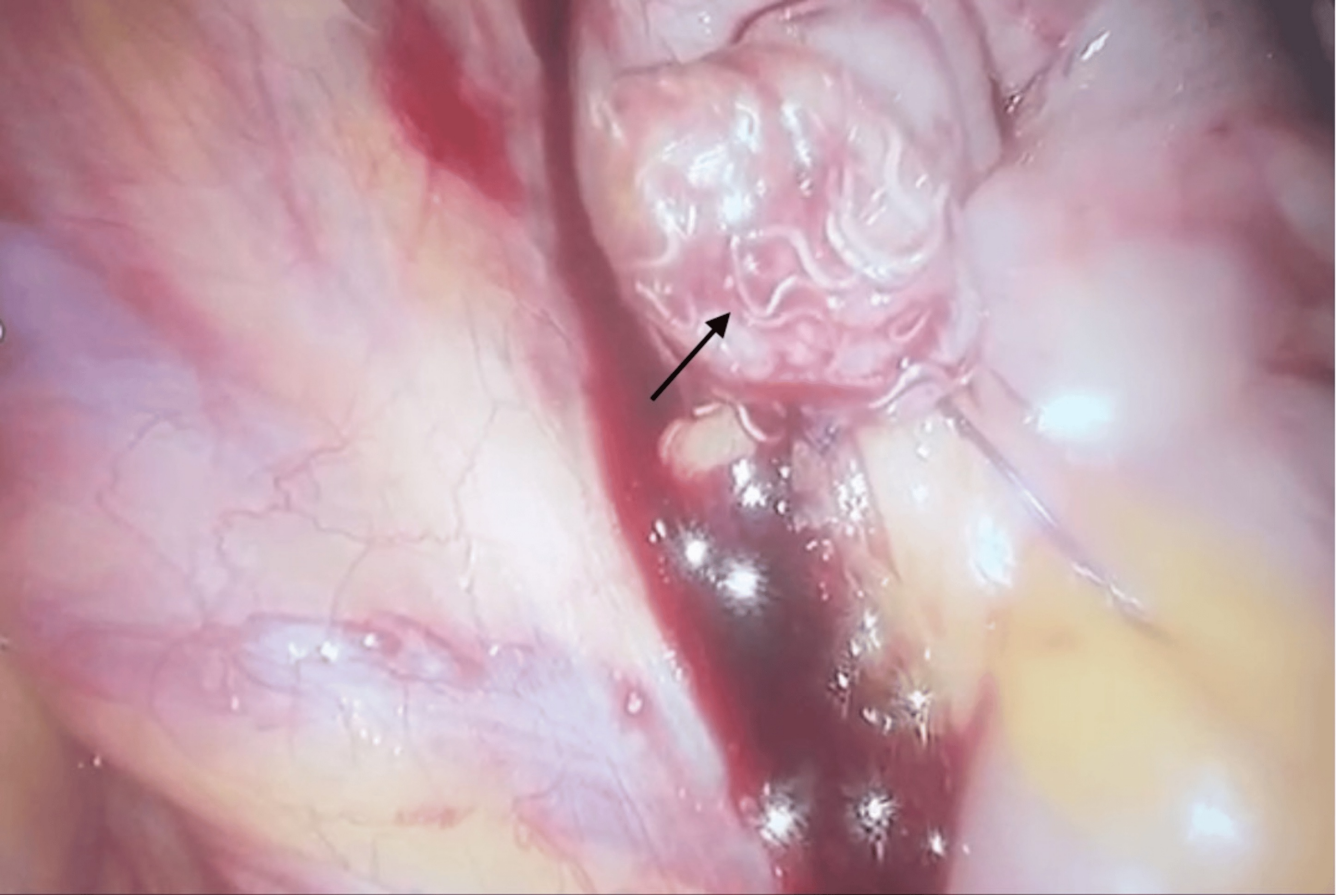
Intraoperative view showing multiple Enterobius vermicularis (pinworms) emerging from the appendiceal stump following laparoscopic appendectomy, confirming the parasitic etiology

The patient underwent a laparoscopic appendicectomy. Intraoperatively, an inflamed appendix was identified. Following application of an endoloop and excision of the appendix, multiple parasitic worms were observed within the appendiceal lumen and stump (Figure 2). Suction and irrigation were performed to remove all visible worms. Additionally, an incidental left inguinal hernia was noted but did not require surgical intervention at this time.

The patient made an uneventful recovery postoperatively and was discharged in stable condition. Empiric antihelminthic treatment with albendazole was initiated, which was administered in two doses, one week apart, as first-line therapy for suspected *E. vermicularis* (pinworm) infection. Family members received prophylactic treatment with the same antihelminthic agent.

**Figure 3:**
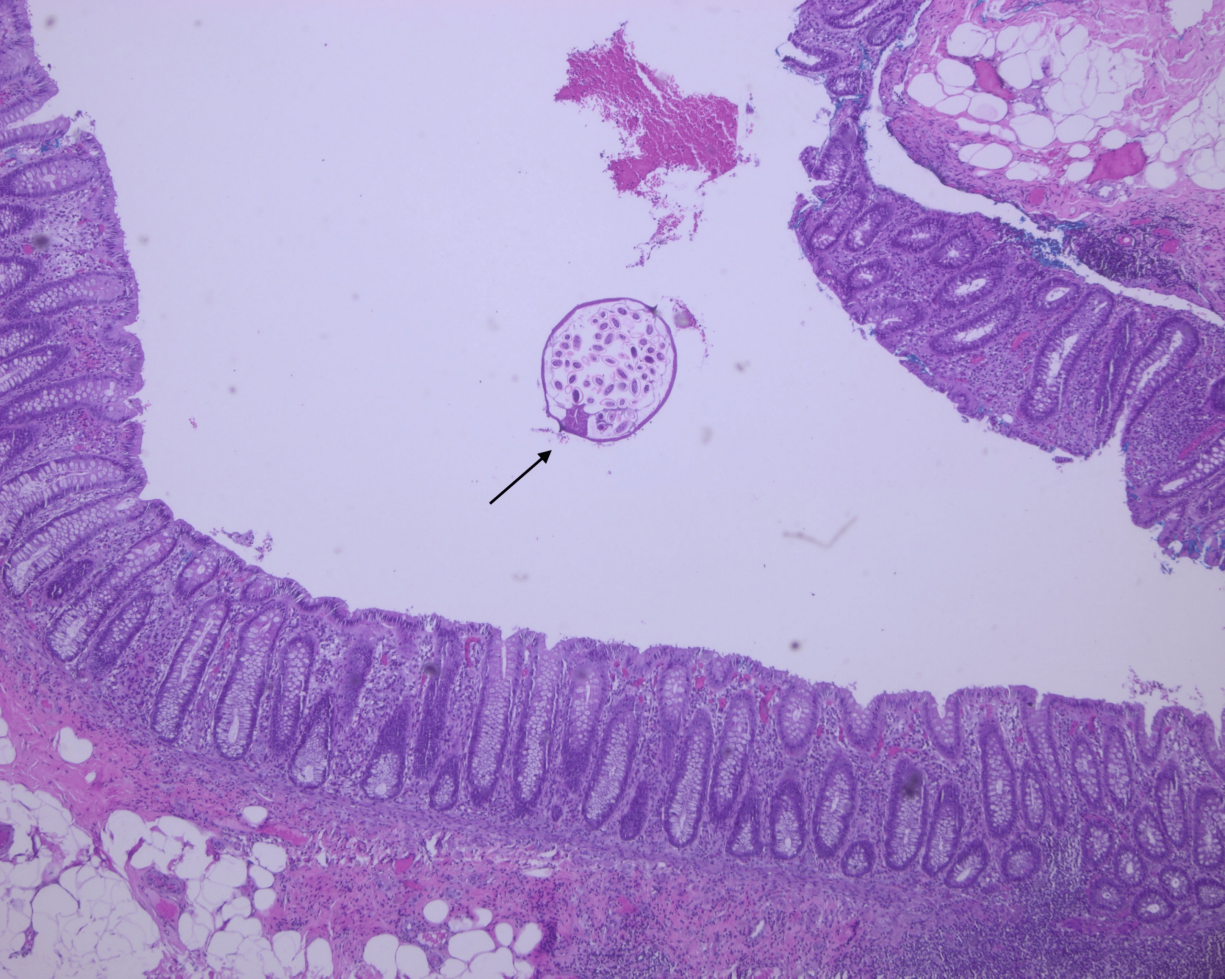
Histopathological examination of the appendix showing E. vermicularis infestation Low-power histopathological image of the appendix lumen containing multiple cross-sections of *E. vermicularis* (H&E, ×40), confirming parasitic infestation.

**Figure 4:**
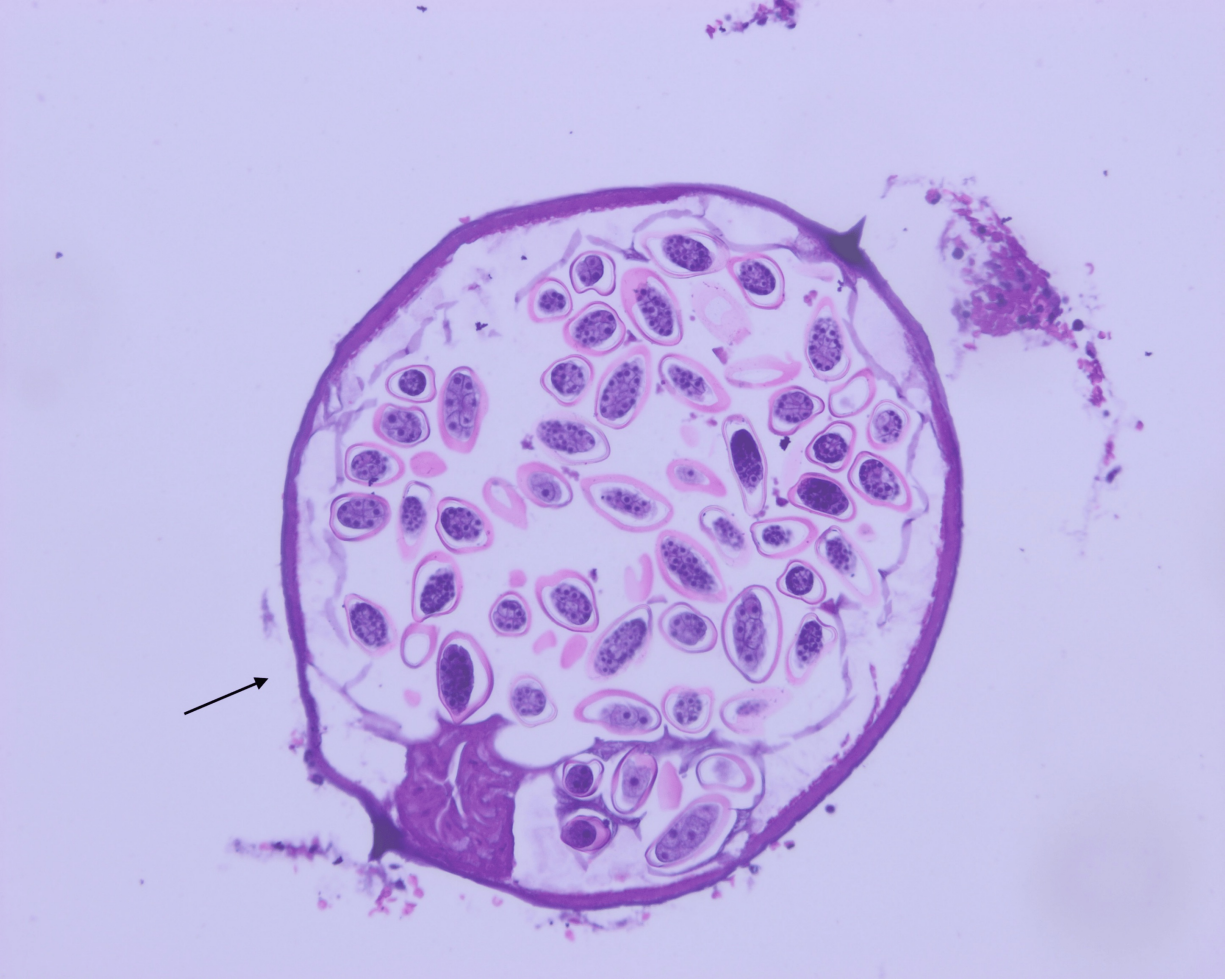
Histopathological examination of the appendix showing E. vermicularis infestation High-power histological view showing the characteristic morphology of *E. vermicularis*, including a thin eosinophilic cuticle and distinctive lateral alae (H&E, ×200).

**Figure 5:**
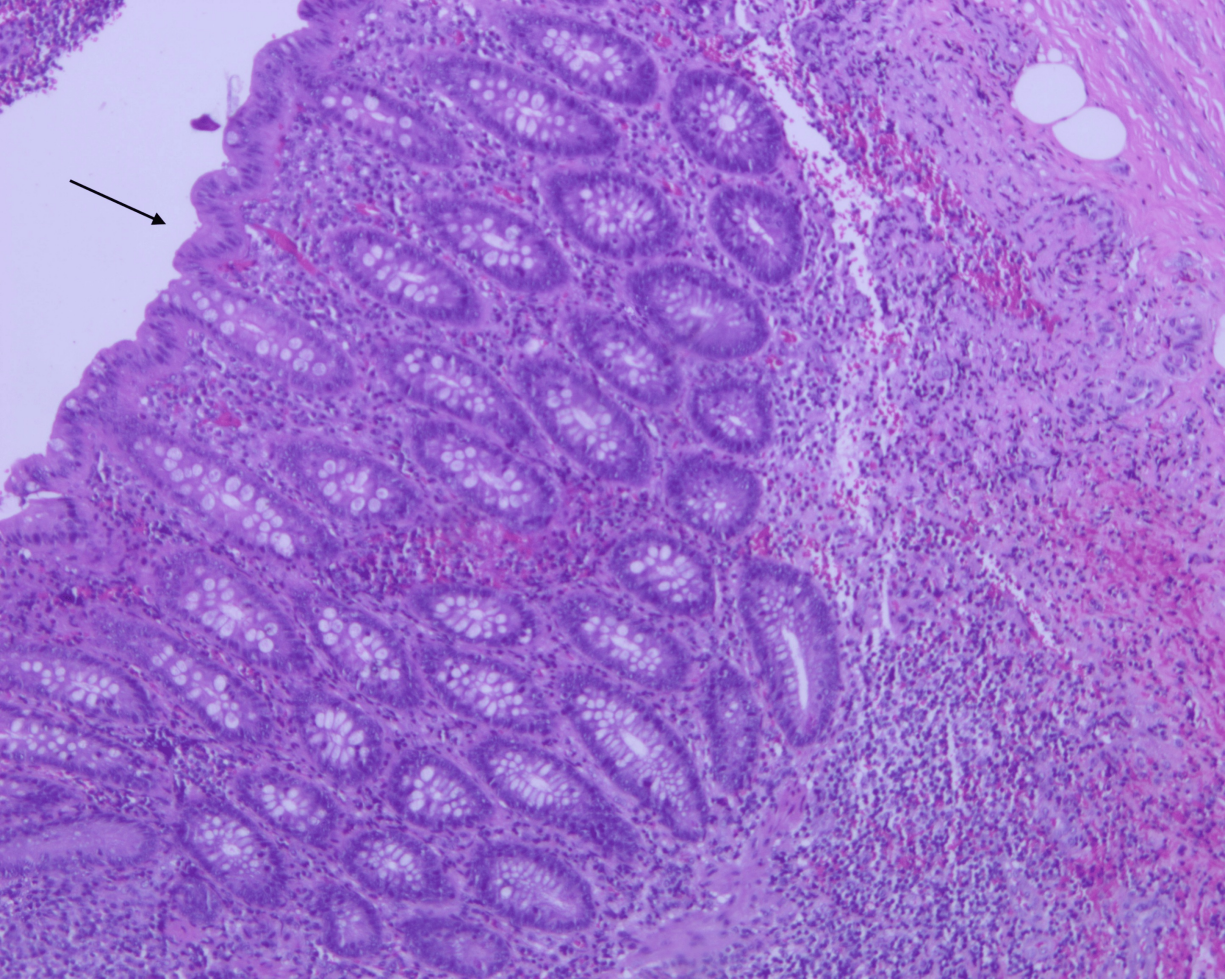
Histopathological examination of the appendix showing E. vermicularis infestation Mid-power histology reveals intact mucosa and mildly expanded lamina propria with reactive lymphoid follicles. No signs of acute inflammation or mucosal ulceration are seen (H&E, ×100).

Histopathology examination of the specimen (Figures 3, 4, 5) shows the appendiceal wall with relatively intact mucosa. The lamina propria was mildly expanded by increased lymphoid follicles and reactive germinal centers. The lumen contained *E. vermicularis*. There was no acute inflammation and no evidence of granuloma, dysplasia, or malignancy.

The patient was followed up at one and four weeks postoperatively as an outpatient in pediatric surgery. At one week, a stool analysis was performed, which was normal following the second dose of albendazole. At four weeks postoperatively, there were no complications related to the surgical procedure.

## Discussion

Appendectomy remains one of the most commonly performed surgical procedures in both pediatric and adult populations. The leading indication for this surgery is acute appendicitis, which typically arises from luminal obstruction due to fecaliths, appendicoliths, or lymphoid hyperplasia [9]. In a retrospective study conducted in Oman among pediatric and adolescent patients undergoing appendectomy for suspected acute appendicitis, parasites were found in 5.5% of cases. Among these, *E. vermicularis* accounted for 51.1%, followed by Schistosoma (9.1%), *Ascaris lumbricoides* (26.1%), *Trichuris trichiura* (8%), and* Taenia* *saginata* (5.7%) [10]. Parasitic infections as a cause of appendicitis are rare, with *E. vermicularis* (pinworm) being the most common cause in such cases.

The typical manifestation of pinworm infection is perianal pruritus, which occurs as gravid females migrate to the anal region at night to deposit eggs. While existing literature acknowledges the involvement of *E. vermicularis* in appendiceal inflammation, some patients exhibit clinical signs of acute appendicitis despite the absence of histological findings, which is known as "appendiceal colic" [11]. Our patient reflected a clinical presentation and imaging findings consistent with early acute appendicitis, yet histopathology revealed no acute inflammation. This underscores the complexity of establishing a causal relationship between *E. vermicularis *and true appendicitis. Nonetheless, *E. vermicularis* can still lead to varying degrees of appendiceal inflammation, ranging from uninflamed appendices to gangrenous or even perforated appendicitis [12]. In our case, despite the absence of raised inflammatory markers, including CRP, neutrophils, and eosinophils, clinical suspicion warranted surgical intervention. The diagnosis was ultimately confirmed intraoperatively when pinworms were visualized emerging from the appendiceal stump after excision. This finding supports the view that parasitic appendicitis cannot be reliably differentiated from other causes of appendicitis preoperatively [13].

Definitive diagnosis of *E. vermicularis* is typically made via the "cellophane tape" test, which detects characteristic eggs in the perianal region. In cases of high parasite burden, stool analysis may also reveal eggs or worms. Once diagnosed, treatment involves administration of anthelmintic agents such as mebendazole, albendazole, or pyrantel embonate. Our patient was treated successfully with albendazole and remained symptom-free following therapy [14].

## Conclusions

*E. vermicularis* can mimic acute appendicitis in children, even in the absence of abnormal laboratory parameters or histological inflammation. In such cases, diagnosis is often confirmed only intraoperatively or through histopathological analysis. This case adds to the limited reports highlighting the diagnostic uncertainty caused by parasitic infections mimicking surgical emergencies. Clinicians should consider *E. vermicularis* in the differential diagnosis of pediatric RIF pain with atypical features. Early surgical intervention and appropriate antiparasitic therapy remain essential for definitive management.

## References

[1] Ferris M, Quan S, Kaplan BS (2017). The global incidence of appendicitis: a systematic review of population-based studies. Ann Surg.

[2] Hammood ZD, Salih AM, Mohammed SH (2019). Enterobius vermicularis causing acute appendicitis, a case report with literature review. Int J Surg Case Rep.

[3] Cook GC (1994). Enterobius vermicularis infection. Gut.

[4] Wiebe BM (1991). Appendicitis and Enterobius vermicularis. Scand J Gastroenterol.

[5] Mardani A, Feizi F, Fakhar M, Beyranvand HB, Farrokhi M, Abbasi M, Asfaram S (2017). Enterobius vermicularis infection among appendectomy specimens in Qom Province, central Iran: a retrospective study. Comp Clin Pathol.

[6] Arca MJ, Gates RL, Groner JI, Hammond S, Caniano DA (2004). Clinical manifestations of appendiceal pinworms in children: an institutional experience and a review of the literature. Pediatr Surg Int.

[7] Lala S, Upadhyay V (2016). Enterobius vermicularis and its role in paediatric appendicitis: protection or predisposition?. ANZ J Surg.

[8] Taghipour A, Olfatifar M, Javanmard E, Norouzi M, Mirjalali H, Zali MR (2020). The neglected role of Enterobius vermicularis in appendicitis: a systematic review and meta-analysis. PLoS One.

[9] Gadiparthi R, Waseem M (2025). Pediatric Appendicitis. https://www.ncbi.nlm.nih.gov/books/NBK441864/.

[10] Zakaria OM, Zakaria HM, Daoud MY (2013). Parasitic infestation in pediatric and adolescent appendicitis: a local experience. Oman Med J.

[11] Fallah E, Dehgani A (2009). A study on Entrobius vermicularis infection in a appendices removed by surgery in Tabriz hospitals. Int J Parasit Dis.

[12] Tayfur M, Balci MG (2019). Pathological changes in appendectomy specimens including the role of parasites: a retrospective study of 2400 cases of acute appendicitis. Niger J Clin Pract.

[13] Akkapulu N, Abdullazade S (2016). Is Enterobius vermicularis infestation associated with acute appendicitis?. Eur J Trauma Emerg Surg.

[14] Wendt S, Trawinski H, Schubert S, Rodloff AC, Mössner J, Lübbert C (2019). The diagnosis and treatment of pinworm infection. Dtsch Arztebl Int.

